# Correlation between integrin alpha 5 expression and the malignant phenotype of transitional cell carcinoma.

**DOI:** 10.1038/bjc.1996.57

**Published:** 1996-02

**Authors:** T. Saito, M. Kimura, T. Kawasaki, S. Sato, Y. Tomita

**Affiliations:** Department of Urology, Niigata University School of Medicine, Japan.

## Abstract

**Images:**


British Journal of Cancer (1996) 73, 327-331
© 1996 Stockton Press All rights reserved 0007-0920/96 $12.00

### Correlation between integrin α5 expression and the malignant phenotype of transitional cell carcinoma

T Saito, M Kimura, T Kawasaki, S Sato and Y Tomita

*Department of Urology, Niigata University School of Medicine, Asahimachi 1, Niigata 951, Japan.*

**Summary** We examined the expression of α1, α2, α3, α4, α5 and β1 integrin on 36 transitional cell cancers (TCCs) in the bladder by immunohistochemistry. Only α2, α3 and β1 were detected on normal transitional cell epithelium, but four TCCs (12.5%) revealed positive staining for α1, seven (19.4%) for α4 and seven (20%) for α5. These altered expressions of integrin α chain were more frequent in histologically higher stage or grade of TCC, and a correlation was found between increased α5 expression and histological stage. α5 was positive in 6 (35.3%) of 17 invasive TCCs whereas only 1 (5.9%) of 17 superficial TCCs. Flow cytometric analysis on bladder cancer cell lines showed that T24 and HT1376, which are undifferentiated TCC cell lines, highly expressed α5 and β1. Also, SCaBER, which is derived from urinary bladder squamous cell cancer and which is recognised as the most malignant phenotype after metaplasia of transitional epithelium, had α5 and β1. However, RT4, which is derived from transitional cell papilloma, showed no expression of α5. Furthermore, reverse transcriptase-polymerase chain reaction (RT-PCR) showed the presence of mRNA of α5 on T24, SCaBER and HT1376, but not on RT4. Taken together, it seems that the presence of α5 integrin might be a more malignant phenotype in transitional cell carcinoma.

**Keywords:** integrin; VLA-5; transitional cell cancer; immunohistochemistry; flow cytometry; reverse transcriptase-polymerase chain reaction

In the process of tumour invasion and metastasis, the interaction between tumour cells and extracellular matrix (ECM), such as laminin, fibronectin and collagens, has a crucial role (Nicolson and Winkelmake, 1975; Horn and Tang, 1992). This interaction is facilitated through adhesion receptors such as integrins. Integrins are a family of cell-surface proteins that mediate cell adhesion to ECM and signal transduction to the cell interior (Hynes, 1990; Albelda, 1993). They are composed of two subunits, α and β, each of which spans the plasma membrane. Distinct α-subunits combine with common or related β-subunits to form functionally distinct receptors.

Integrin distributions have been studied in a number of tissues, including malignant tumours. Recent studies showed that patterns of integrin expression on tumour cells were different compared with normal counterparts and suggested that altered integrin expression may contribute to the invasive or metastatic phenotype. For example, the loss of integrin expression was reported in epithelial neoplasms, such as carcinomas of the breast, colon, pancreas and skin (Pignatelli *et al.*, 1990; Zutter *et al.*, 1990; Hall *et al.*, 1991; Stamp and Pignatelli, 1991). On the other hand, up-regulation of αVβ3 was described in malignant melanoma and glioblastoma multiforme, and acquisition of α4β1 has been described in malignant melanoma and renal cell carcinoma (Cheresh *et al.*, 1989; Gladson and Cheresh, 1991; Tomita *et al.*, 1995). In transitional cell carcinoma (TCC), progressive loss of α2 integrin expression from normal urothelial cells through invasive cancers was reported and some α5 integrin was expressed on high stage TCC (Leibert *et al.*, 1994).

VLA-5 (α5β1 integrin) is a fibronectin receptor whose expression is often reduced in tumour cells (Plantefaber and Hynes, 1989). In addition, increasing the expression of the α5β1 integrin by gene transfer decreases the formation of tumours on Chinese hamster ovary cells, suggesting the presence of VLA-5 on tumour cells might be a disadvantage for their proliferation (Giancotti and Ruoslahti, 1990). Indeed, after transfection, they showed less migratory, reacquired features of normal growth control in culture, resulting in lost ability to form tumours when injected subcutaneously into nude mice (Giancotti and Ruoslahti, 1990). Several other studies, including some on human tumour cells, confirmed the correlation between low VLA-5 expression and malignant transformation or higher malignant potential (Varner *et al.*, 1992; Witkowski *et al.*, 1993).

The expression of VLA-5 on high stage TCC in a previous report seemed curious. Therefore, we focused our interest on the expression of VLA-5 on TCC and the normal transitional cell and its mRNA. We examined the expression of integrins on 36 TCCs and six normal transitional cells by immunohistochemistry. Flow cytometric analysis and RT-PCR for VLA-5 or mRNA of α5 integrin on bladder cancer cell lines and normal transitional cell line were also performed. We showed that the presence of VLA-5 might indicate a more malignant phenotype, as for TCC.

### Materials and methods

### *Tissue specimens*

Tumour specimens were obtained from 36 patients (25 males and 11 females) who had undergone total cystectomy or transurethral resection for bladder cancer. The mean age at the time of operation was 67.9 years, ranging from 41 to 84 years. Six specimens of normal urinary bladder epithelium were collected from a histologically unaffected portion of the bladder. Tissue samples were embedded in an optimum cold temperature compound (Miles Laboratories, Naperville, IL, USA) and were quickly frozen in isopentane, precooled in dry ice acetone. These blocks were stored at −80°C until 5 μm serial sections were cut using a cryostat. Histological examination was performed on haematoxylin and eosin-stained tissue sections. Tumours were graded and staged according to the criteria of the World Health Organization.

### *Reagents*

Monoclonal antibodies (MAbs) used in this study were as follows: TS2/7 against α1, P1E6 against α2, P1B5 against α3, P4G9 against α4, P1D6 against α5, 4B4 against β1. Polyclonal anti-α5 antibody AB1928 was also used. These MAbs were purchased from Telious Pharmaceuticals (San Diego, CA, USA), except for 4B4 which was purchased from Corter (Hialeah, FL, USA) and AB1928, which was

Correspondence: T Saito
Received 13 March 1995; revised 22 August 1995; accepted 11 September 1995

purchased from Chemicon International (Temecula, CA, USA). Optimal dilution for each antibody was determined by staining specimens of lymph nodes obtained during nephrectomy for renal cell carcinoma without tumour metastasis.

*Immunoperoxidase staining*

Immunoperoxidase staining was performed using the streptavidin–biotin bridge technique as described previously (Tomita *et al.*, 1990). Briefly, serial sections were air dried and fixed in cold acetone. After rehydration with phosphate-buffered saline (PBS), sections were incubated in PBS containing 20% normal sheep serum (Antibodies, Davis, CA, USA) for 30 min, and endogenous biotin was blocked using an endogenous biotin blocking kit (Vector Laboratories, Burlingame, CA, USA). They were then incubated with mouse MAbs for 60 min, followed by incubation with biotinylated sheep anti-mouse immunoglobulin (Amersham International, Amersham, Bucks, UK) in PBS containing 20% human type AB serum (Biological Speciality, Lansdale, PA). Subsequently, they were incubated with streptavidin–peroxidase (Amersham) for 15 min. Each step was followed by washing in PBS with three changes of buffer. Finally, the sections were immersed in 0.05 mol $l^{-1}$ Tris-HCl buffer containing 0.05% diaminobenzene and 0.01% hydrogen peroxide for 4–20 min to visualise the reaction products. Specimens were counterstained in Meayer's haematoxylin and mounted after dehydrating in graded ethanol and xylene. Tumours were considered as positive when positive tumour cells were observed in the specimen, although the tumour tissue showed various staining pattern when reacted with anti-$\alpha$5 MAb. Statistical analysis was done by using the chi-square test.

*Flow cytometric analysis of bladder cancer cell lines*

To investigate differences in $\alpha$5 expression among bladder cancer cell lines with various characteristics, we used four established bladder cancer cell lines and a normal transitional cell line obtained from the American Type Culture Collection (ATCC). Three cancer cell lines, T24 (Bubenik *et al.*, 1973), SCaBER (O'Toole *et al.*, 1976) and HT1376 (Rasheed *et al.*, 1977) were derived from high-grade bladder cancers; one, RT4 (Rigby and Franks, 1970), was derived from papilloma and HTB160 was derived from normal fetal bladder cells. These cell lines were cultured in 3055 (Coaster, CA, USA) 25 $cm^2$ tissue culture flasks in complete medium (RPMI-1640 medium containing 10% fetal calf serum). For flow cytometric analysis, cells were stained by the indirect immunofluorescence method as described previously (Tomita *et al.*, 1990). Briefly, tumour cell suspensions were prepared by treatment with 0.125% trypsin and 0.02% EDTA. Tumour cells were reacted with anti-$\alpha$5 or $\beta$1 MAb in PBS supplemented with 2% fetal calf serum and 0.02% sodium azide for 30 min at 4°C. After washing twice by centrifugation, cells were incubated with fluorescein isothiocyanate-conjugated goat anti-mouse Ig (Tago, Burlingame, CA, USA) for 30 min at 4°C. Subsequently, the cells were washed three times and analysed by flow cytometry (FACScan, Becton-Dickinson).

*Reverse transcription of RNA followed by the polymerase chain reaction (RT–PCR) analysis*

Total RNA was isolated from cell lines using RNAzol (Biotecx Laboratories, USA) according to the instructions of the manufacturers. Total RNA (10 $\mu$g) was used for cDNA synthesis. First-strand cDNA solution (2 $\mu$l) was then used for PCR, with primers designed to amplify a 1421 bp $\alpha$5 cDNA sequence from bp 1625 to bp 3046 (sense primer sequence: 5'-AACAGGATGGCTAGGATGAT, antisense primer sequence: 5'-ACAAGTTGCTGACTCCATTG). PCR was performed in 50 $\mu$l buffer (50 mmol $l^{-1}$ potassium chloride; 10 mmol $l^{-1}$ Tris-HCl, pH 8.4; 1.5 mmol $l^{-1}$ magnesium chloride and 200 mg $ml^{-1}$ gelatin), with 200 mmol $l^{-1}$ of each dNTP, 2.5 mmol $l^{-1}$ of each oligonucleotide primer, and 2.5 U of *Taq* DNA polymerase (Boehringer Mannheim, Germany). Thirty-five cycles of 1 min denaturation at 94°C, 1 min annealing at 60°C, and a 1.5 min extension step at 72°C were performed. At the end of the 35 cycles, an additional 10 min extension step at 72°C was added. Subsequently, to confirm the specificity of this product, nested PCR was performed using 5 $\mu$l of PCR product with primers designed to amplify a 1268 bp $\alpha$5 cDNA sequence from bp 1657 to bp 2925 (sense primer sequence: 5'-TCAGGGATCCAACTTCAGCTGGACTGGCAGAAGCA, antisense primer sequence: 5'-GATCGAATTCGGGCATCTTCAGGGCTTTGTACACA).

### Results

In situ *expressions of integrins on TCCs*

When normal transitional cells were stained with anti-integrin MAbs, constitutional expressions of $\alpha$2, $\alpha$3 and $\beta$1 were detected. However, $\alpha$1, $\alpha$4 and $\alpha$5 were negative for all six normal transitional cell specimens. On the contrary, four TCCs (12.5%) revealed positive staining for $\alpha$1, 7 (19.4%) for $\alpha$4 and 7 (20%) for $\alpha$5 (Figure 1). Three TCCs (8.3%) showed decreased expression of $\alpha$2. These altered expressions of integrin $\alpha$ chain were more frequent in histologically higher stage or higher grade of TCC (Table I). Although statistically not significant, a correlation was found between increased $\alpha$5 expression and the histological stages. $\alpha$5 was positive in 6 (35.3%) of 17 invasive TCCs but only 1 (5.9%) of 17 superficial TCCs (Table II).

**Figure 1** Immunohistochemical staining for $\alpha$5 integrin. (**a**) Grade 3 invasive (pT4) bladder TCC positively stained with anti-$\alpha$5 integrin. (**b**) Grade 1 non-invasive (pT1) bladder TCC was negative for $\alpha$5. Scale bars = 75 $\mu$m.

**Table I** Clinical and histological features and expression of integrins on TCC

| *No.* | *Age* | *Sex* | *T-stage* | *Grade* | $\alpha1$ | $\alpha2$ | $\alpha3$ | $\alpha4$ | $\alpha5$ | $\beta1$ |
|---|---|---|---|---|---|---|---|---|---|---|
| 1 | 62 | M | 2 | 2 | − | + | + | − | − | + |
| 2 | 52 | M | 4 | 3 | − | + | + | + | − | + |
| 3 | 59 | M | 1 | 2 | − | + | + | − | − | + |
| 4 | 58 | M | 1 | 2 | − | + | + | − | − | + |
| 5 | 72 | F | 1 | 3 | − | + | + | − | − | + |
| 6 | 73 | M | 4 | 3 | − | + | + | − | − | + |
| 7 | 65 | M | 3 | 3 | − | + | + | − | − | + |
| 8 | 62 | M | 2 | 3 | − | + | + | − | − | + |
| 9 | 73 | M | 3 | 3 | − | + | + | + | − | + |
| 10 | 74 | M | 1 | 3 | + | + | + | + | − | + |
| 11 | 69 | F | 3 | 2 | − | + | + | − | ND | + |
| 12 | 67 | F | 3 | 3 | − | + | + | − | + | + |
| 13 | 79 | M | 4 | 3 | − | + | + | − | − | + |
| 14 | 70 | M | 1 | 3 | − | + | + | − | − | + |
| 15 | 60 | M | 4 | 3 | − | + | + | + | − | + |
| 16 | 72 | M | 2 | 3 | − | + | + | − | − | + |
| 17 | 72 | F | 1 | 3 | − | + | + | − | − | + |
| 18 | 67 | F | 2 | 2 | − | + | + | − | − | + |
| 19 | 72 | M | 1 | 2 | − | + | + | − | − | + |
| 20 | 60 | F | 3 | 3 | + | + | + | − | + | + |
| 21 | 41 | M | 2 | 2 | − | − | + | + | + | + |
| 22 | 76 | F | 3 | 3 | − | + | + | − | + | + |
| 23 | 77 | M | 4 | 2 | − | + | + | − | + | + |
| 24 | 72 | M | 2 | 2 | − | + | + | − | − | + |
| 25 | 75 | M | 3 | 3 | + | + | + | − | + | + |
| 26 | 76 | M | 1 | 2 | − | + | + | + | − | + |
| 27 | 65 | F | 1 | 3 | − | + | + | + | − | + |
| 28 | 41 | F | 1 | 2 | − | + | + | − | − | + |
| 29 | 74 | M | 1 | 3 | − | − | + | − | − | + |
| 30 | 68 | M | 1 | 2 | − | + | + | − | − | + |
| 31 | 58 | M | 1 | 1 | − | + | + | − | − | + |
| 32 | 68 | M | 1 | 1 | + | + | + | − | + | + |
| 33 | 69 | F | a | 1 | ND | + | + | − | − | + |
| 34 | 84 | M | 1 | 1 | ND | + | + | − | − | + |
| 35 | 82 | M | a | 1 | ND | + | + | − | − | + |
| 36 | 82 | F | 1 | 2 | ND | + | + | − | − | + |

−, Negative; +, positive; ND, not done

**Table II** Correlation between $\alpha5$ expression on bladder cancer and T-stage

| | *$\alpha5$ expression on TCC* | |
|---|---|---|
| | − | + |
| Ta, T1 | 16 | 1 |
| T2–T4 | 11 | 6 |

*Flow cytometric analysis of the expression of $\alpha5$ and $\beta1$ integrins on human TCC cell lines*

Results of immunohistochemical staining against $\alpha5$ prompted us to investigate its expression on bladder cancer cell lines and the transitional cell line. Flow cytometric analyses showed $\alpha5$ molecules on T24 and HT1376, which are TCC cell lines with malignant phenotype, and SCaBER, which is a bladder SCC cell line, but not on RT4 derived from benign papilloma. However, HTB160, which is a normal TCC line, showed the presence of $\alpha5$. All of them showed positive staining against $\beta1$ (Figure 2).

*Detection of $\alpha5$ mRNA by RT–PCR*

To confirm the presence of $\alpha5$ mRNA, we performed nested RT–PCR using two sets of primers. Integrin $\alpha5$ cDNA fragments of the expected size could be amplified from T24, SCaBER, HT1376 and HTB160 mRNA. However, RT–PCR for RT4 mRNA did not show any bands (Figure 3).

### Discussion

In order to investigate the alteration of integrin expression of TCC, we immunohistochemically examined $\alpha1$, $\alpha2$, $\alpha3$, $\alpha4$, $\alpha5$ and $\beta1$ integrin on bladder TCC as well as normal transitional epithelium. When normal transitional cell epithelium was stained with the panel of MAbs, only $\alpha2$, $\alpha3$ and $\beta1$ were positive. On the other hand, some TCCs expressed $\alpha1$, $\alpha4$ and $\alpha5$, and these altered expressions of integrins were observed relatively frequently in the higher grade and/or stage tumours. These results suggest that increased expression of $\alpha1$, $\alpha4$ and $\alpha5$ might change the character of TCC to ECM and facilitate tumour invasion or metastasis. Also, a significant correlation was found only between increased $\alpha5$ expression and the histological stage. Leibert *et al.* (1994) also showed that $\alpha5$ integrin was expressed on high-stage TCC but not on low-stage tumours. However, $\alpha5$ was detected on TCC in a rather smaller number of tumours than in the present study, and there was no positive staining of $\alpha4$. These discrepancies may be explained by the difference in staining methods and/or MAbs used. We might use a possibly more sensitive staining procedure, amplifying positive staining by the streptavidin–biotin system, which we used in other studies (Tomita *et al.*, 1990, 1993).

A member of the integrin $\beta1$ subfamily, VLA-5 ($\alpha5\beta1$), is a fibronectin receptor, and its expression is often reduced in tumour cells (Plantefaber and Hynes, 1989). In addition, increase in $\alpha5\beta1$ expression by gene transfection decreases the formation of tumours on Chinese hamster ovary cells, suggesting that the presence of VLA-5 on cells might be a disadvantage for tumour cell proliferation by transducing growth-inhibitory stimuli from fibronectin (Schreiner *et al.*, 1991). Indeed, after transfection, they showed less migration and reacquired the features of normal growth control in culture, resulting in loss of the ability to form tumours when injected subcutaneously into nude mice (Giancotti and Ruoslahti, 1990). Several other studies, including human tumour cells, have confirmed the correlation between low

**Figure 2** Flow cytometric analysis for α5 and β1 chain expression on human TCC cell lines and transitional cell line. Both expressions were examined on T24, HT1376, SCaBER, RT4 and HTB160. RT4 did not express α5 integrin.

**Figure 3** Detection of α5 mRNA by nested RT–PCR. (**a**) RT–PCR analysis of α5 mRNA. T24 (lane 5), HT1376 (lane 4), SCaBER (lane 3) and HTB160 (lane 1) were positive. RT4 (lane 2) was negative. M, marker. (**b**) Nested PCR. T24 (lane 5), HT1376 (lane 4), SCaBER (lane 3) and HTB160 (lane 1) were positive. RT4 (lane 2) was negative.

VLA-5 expression and malignant transformation or a higher malignancy (Varner *et al.*, 1992; Witkowski *et al.*, 1993).

The present study on TCC, however, showed a more frequent expression of α5 and β1 chain on TCCs of higher grade and histopathological stage. In addition, examination of normal transitional cells showed no α5 staining. Compatible with the results of immunohistochemistry on TCC, flow cytometric analysis on cultured bladder cancer cell lines showed that T24, SCaBER and HT1376, which are undifferentiated TCC or SCC cell lines said to have a higher malignant character, revealed high expression of α5 and β1, whereas RT4, which is derived from transitional cell papilloma and preserves a well-differentiated character, showed no expression of α5. SCC of the urinary bladder is recognised as the most malignant phenotype after metaplasia of transitional epithelium. Furthermore, RT–PCR showed the presence of mRNA on T24, SCaBER and HT1376, but not on RT4. Taken together, it seems more likely that, as for TCC, the presence of VLA-5 might be a more malignant phenotype. There have been some reports of the presence of VLA-5 with malignant phenotype as in the present study (Terpe *et al.*, 1993; Leibert *et al.*, 1994). Interestingly, HTB160, which is a normal TCC line, showed positive staining against α5. However, HTB160 was developed from fetal bladder. It is possible that the expression of integrin is different between adult and fetal bladder because its expression is reported to be different between adult and fetal kidney (Korhonen *et al.*, 1990).

In conclusion, we have shown the significance of the expression of integrins on bladder transitional cell cancer, especially the correlation between VLA-5 and malignant behaviour of TCC cells. Although the functional aspect of these results are still unclear, integrins, especially VLA-5, might have important roles in the invasion and metastasis of transitional cell cancer.

**Acknowledgements**
The authors thank Dr T Tanikawa, (Department of Urology, Niigata University School of Medicine) for his advice on tumour pathology and Mr T Kashiwaya, (Department of Urology, Niigata University School of Medicine) for his skilful technical assistance.

### References

ALBELDA SM. (1993). Role of integrins and other cell adhesion molecules in tumor progression and metastasis. *Lab. Invest.*, **68**, 4–17.

BUBENIK J, BARESOVA M, VIKLICKY V, JAKOUBKOVA J, SAINEROVA H AND DONNER J. (1973). Established cell line of urinary bladder carcinoma (T24) containing tumour specific antigen. *Int. J. Cancer*, **11**, 765–773.

CHERESH D, SMITH J, COOPER H AND QUARANTA V. (1989). A novel vitronectin receptor integrin ($\alpha V\beta X$) is responsible for distinct adhesion properties of carcinoma cells. *Cell*, **57**, 59–69.

GLADSON CL AND CHERESH DA. (1991). Glioblastoma expression of vitronection and the $\alpha V\beta 3$ integrin. *J. Clin. Invest.*, **88**, 1924–1932.

GIANCOTTI FG AND RUOSLAHTI E. (1990). Elevated levels of the $\alpha 5\beta 1$ fibronection receptor suppress the transformed phenotype of Chinese hamster ovary cells. *Cell*, **60**, 849–859.

HALL PA, COATES P, LEMOINE NR AND HORTON MA. (1991). Characterization of integrin chains in normal and neoplastic human pancreas. *J. Pathol.*, **165**, 33–41.

HORN KV AND TANG DG. (1992). Adhesion molecules and tumor cell interaction with endothelium and subendothelial matrix. *Cancer Met. Rev.*, **11**, 353–375.

HYNES RO. (1990). Integrins: a family of cell surface receptors. *Cell*, **48**, 549–554.

NICOLSON GL AND WINKELMAKE JL. (1975). Organ specificity of blood-borne tumor metastasis determined by cell adhesion? *Nature*, **255**, 230–232.

KORHONEN M, YLANNE J, LAITINEN L AND VIRTANEN I. (1990). The $\alpha 1-\alpha 6$ subunits of integrins are characteristically expressed in distinct segments of developing and adult human nephron. *J. Cell. Biol.*, **111**, 1245–1254.

LEIBERT M, WASHINGTON R, STEIN J, WEDEMEYER G AND GROSSMAN HB. (1994). Expression of the VLA $\beta 1$ integrin family in bladder cancer. *Am. J. Pathol.*, **144**, 1016–1022.

O'TOOLE C, NAYAK S, PRICE Z, GILBERT WH AND WAISMAN J. (1976). A cell line (SCaBER) derived from squamous cell carcinoma of the human urinary bladder. *Int. J. Cancer*, **17**, 707–714.

PIGNATELLI M, SMITH MEF AND BODMER WF. (1990). Low expression of collagen receptors in moderate and poorly differentiated adenocarcinomas. *Br. J. Cancer*, **61**, 636–638.

PLANTEFABER LC AND HYNES RO. (1989). Changes in integrin receptors on ontogenically transformed cells. *Cell*, **56**, 281–290.

RASHEED S, GARDNER MB, RONGEY RW, NELSON-REES WA AND ARNSTEIN P. (1977). Human bladder carcinoma: characterization of two new tumor cell lines and search for tumor viruses. *J. Natl Cancer Inst.*, **58**, 881–890.

RIGBY CC AND FRANKS LM. (1970). A human tissue culture cell line from a transitional cell tumor of the urinary bladder: growth, chromosome pattern and ultrastructure. *Br. J. Cancer*, **24**, 746–754.

SCHREINER C, FISHER M, HUSSEIN S AND JULIANO RL. (1991). Increased tumorigenicity of fibronectin receptor deficient Chinese Hamster ovary cell variants. *Cancer Res.*, **51**, 1738–1740.

STAMP GWH AND PIGNATELLI M. (1991). Distribution of $\beta 1$, $\alpha 1$, $\alpha 2$ and $\alpha 3$ integrin chains in basal cell carcinomas. *J. Pathol.*, **163**, 307–313.

TERPE HJ, TAJROBEHKAR K, GUNTHERT U AND ALTMANNSBERGER M. (1993). Expression of cell adhesion molecules alpha-2, alpha-5 and alpha-6 integrin, E-cadherin, N-CAM and CD-44 in renal cell carcinomas. An immunohistochemical study. *Virchows Archiv. A Pathol. Anat.*, **422**, 219–224.

TOMITA Y, NISHIYAMA T, FUJIWARA M AND SATO S. (1990). Immunohistochemical detection of major histocompatibility complex antigens and quantitative analysis of tumour-infiltrating mononuclear cells in renal cell cancer. *Br. J. Cancer*, **62**, 354–359.

TOMITA Y, WATANABE H, KOBAYASHI H, NISHIYAMA T, TSUJI S, IMAI K, ABO T, FUJIWARA M AND SATO S. (1993). Expression of intercellular adhesion molecule-1 on transitional cell cancer: Possible significance in immunity against tumor cells. *Am. J. Pathol.*, **143**, 191–198.

TOMITA Y, SAITO T, SAITO K, OITE T, SHIMIZU F AND SATO S. (1995). Significance of VLA-4 ($\alpha 4\beta 1$) for hematogenous metastasis of renal cell cancer. *Int. J. Cancer*, **60**, 1–6.

VARNER JA, FISHER MH AND JULIANO RL. (1992). Ectopic expression of integrin alpha 5 beta 1 suppresses in vitro growth and tumorigenicity of human colon carcinoma cells. *Mol. Biol. Cell*, **3**, 232a.

WITKOWSKI CM, RABINOVITZ I, NAGLE RB, AFFINITO KSD AND CRESS AE. (1993). Characterization of integrin subunits, cellular adhesion and tumorigenicity of four human prostate cell lines. *J. Cancer Res. Clin. Oncol.*, **119**, 637–644.

ZUTTER MM, MAZOUJIAN G AND SANTORO SA. (1990). Decreased expression of integrin adhesive protein receptors in adenocarcinomas of the breast. *Am. J. Pathol.*, **137**, 863–870.


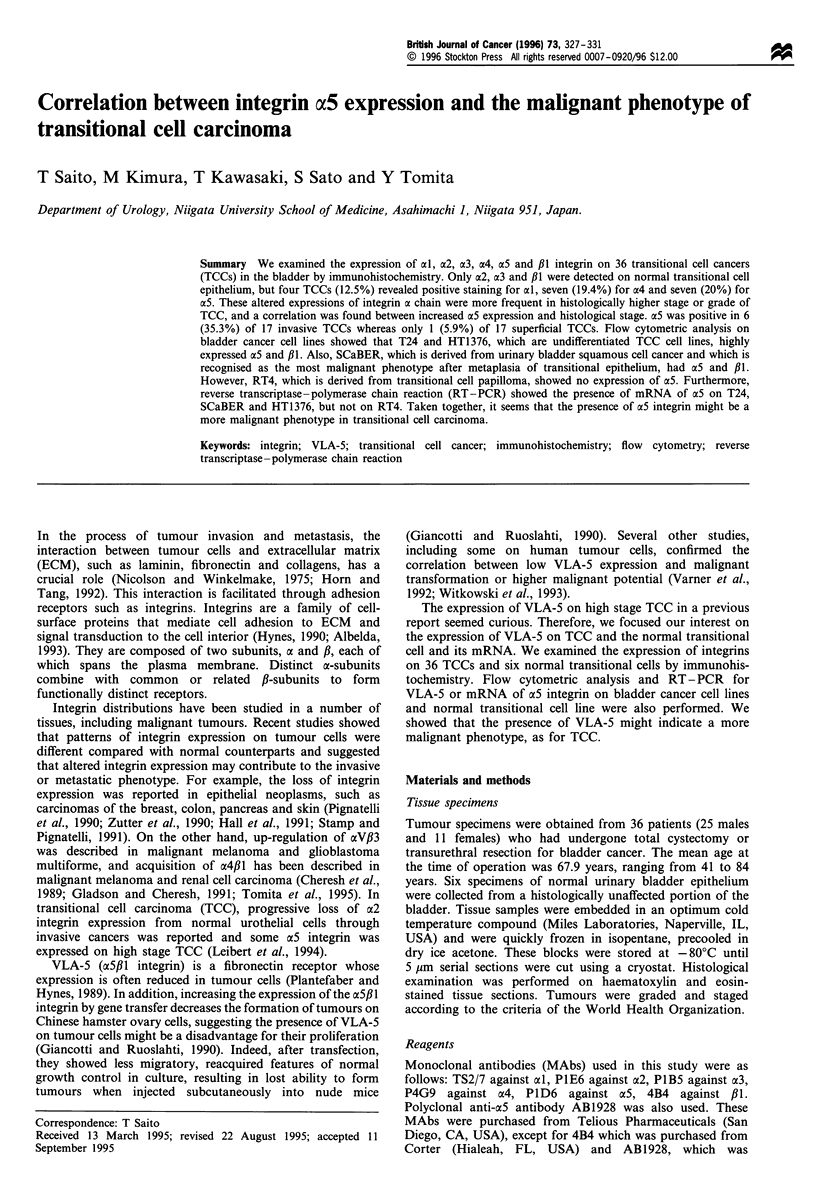

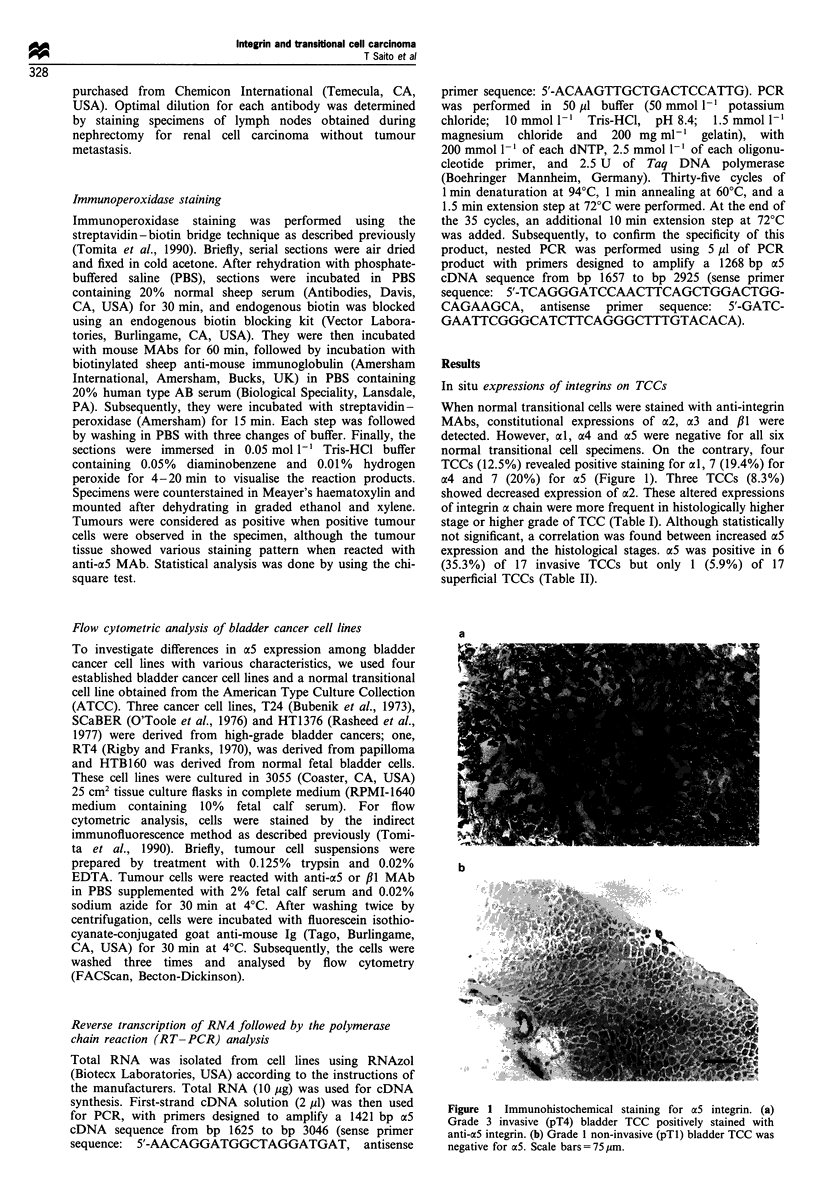

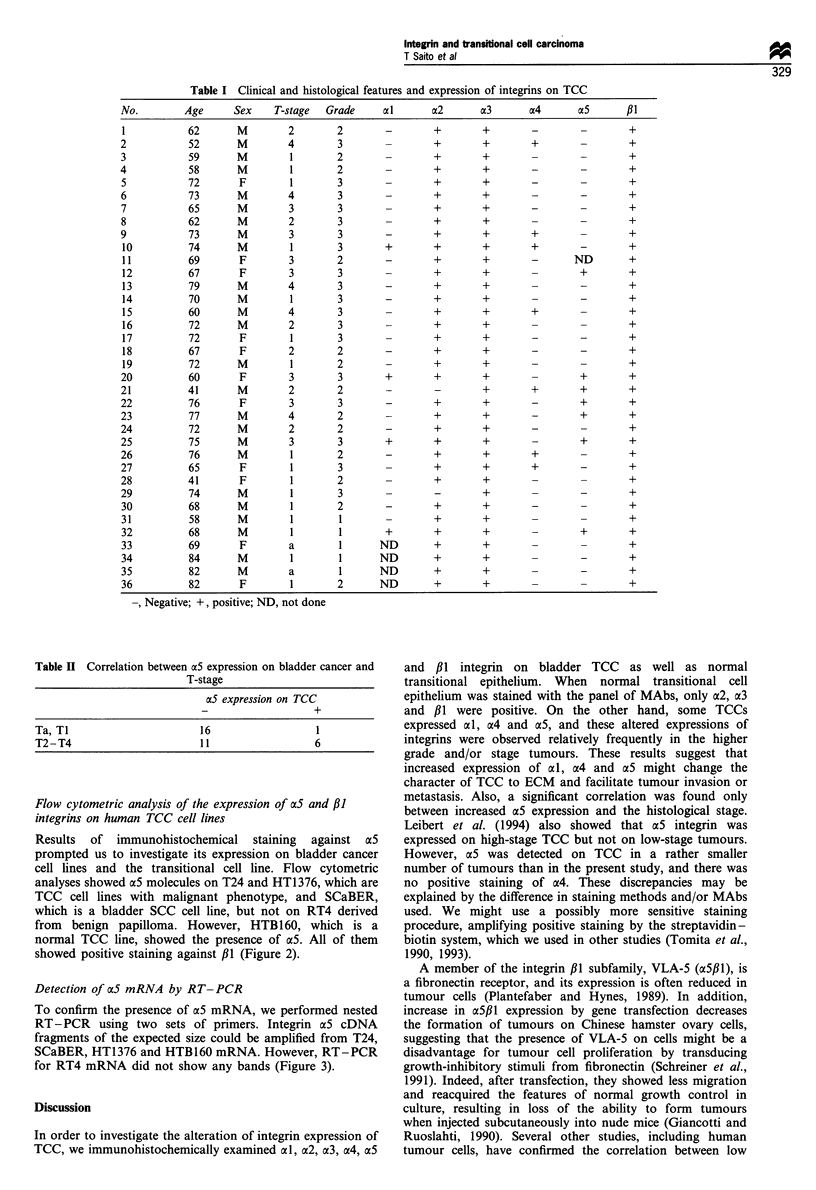

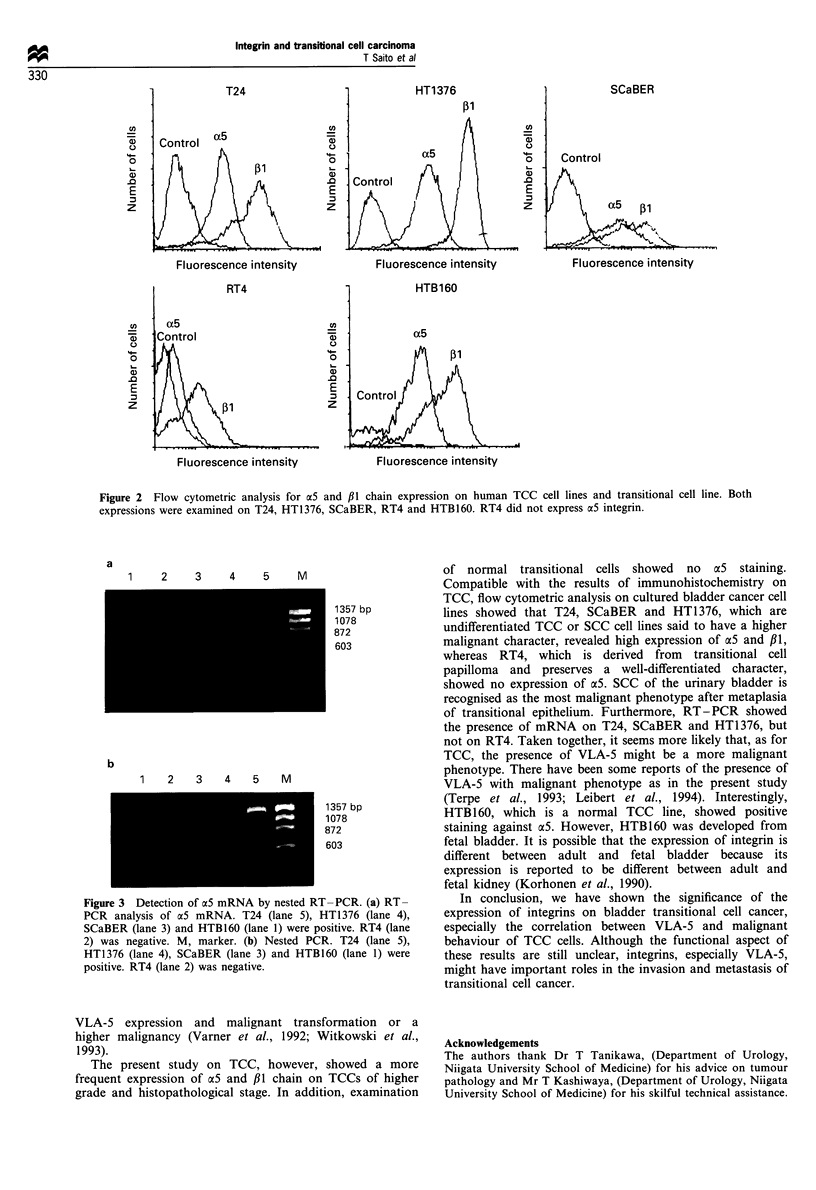

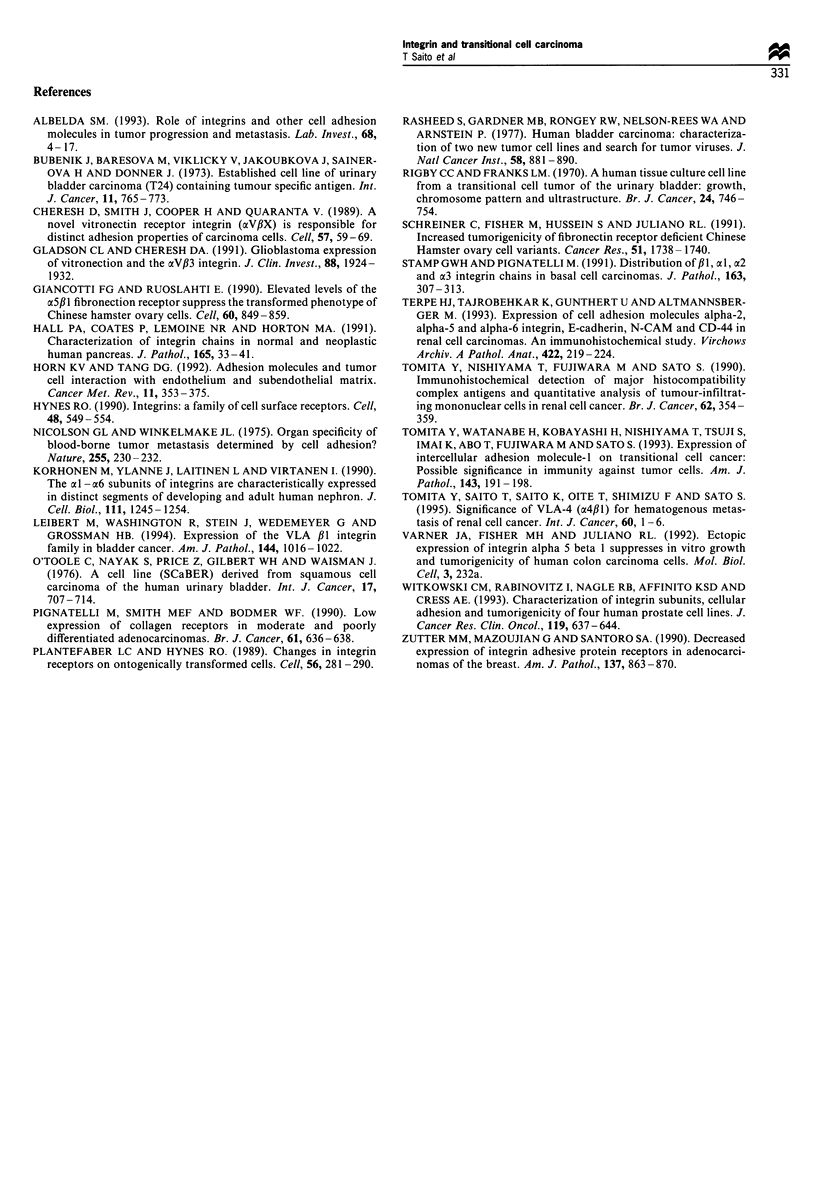


## References

[OCR_00543] Albelda S. M. (1993). Role of integrins and other cell adhesion molecules in tumor progression and metastasis.. Lab Invest.

[OCR_00548] Bubeník J., Baresová M., Viklický V., Jakoubková J., Sainerová H., Donner J. (1973). Established cell line of urinary bladder carcinoma (T24) containing tumour-specific antigen.. Int J Cancer.

[OCR_00556] Cheresh D. A., Smith J. W., Cooper H. M., Quaranta V. (1989). A novel vitronectin receptor integrin (alpha v beta x) is responsible for distinct adhesive properties of carcinoma cells.. Cell.

[OCR_00566] Giancotti F. G., Ruoslahti E. (1990). Elevated levels of the alpha 5 beta 1 fibronectin receptor suppress the transformed phenotype of Chinese hamster ovary cells.. Cell.

[OCR_00561] Gladson C. L., Cheresh D. A. (1991). Glioblastoma expression of vitronectin and the alpha v beta 3 integrin. Adhesion mechanism for transformed glial cells.. J Clin Invest.

[OCR_00569] Hall P. A., Coates P., Lemoine N. R., Horton M. A. (1991). Characterization of integrin chains in normal and neoplastic human pancreas.. J Pathol.

[OCR_00574] Honn K. V., Tang D. G. (1992). Adhesion molecules and tumor cell interaction with endothelium and subendothelial matrix.. Cancer Metastasis Rev.

[OCR_00581] Hynes R. O. (1987). Integrins: a family of cell surface receptors.. Cell.

[OCR_00590] Korhonen M., Ylänne J., Laitinen L., Virtanen I. (1990). The alpha 1-alpha 6 subunits of integrins are characteristically expressed in distinct segments of developing and adult human nephron.. J Cell Biol.

[OCR_00594] Liebert M., Washington R., Stein J., Wedemeyer G., Grossman H. B. (1994). Expression of the VLA beta 1 integrin family in bladder cancer.. Am J Pathol.

[OCR_00583] Nicolson G. L., Winkelhake J. L. (1975). Organ specificity of blood-borne tumour metastasis determined by cell adhesion?. Nature.

[OCR_00601] O'Toole C., Nayak S., Price Z., Gilbert W. H., Waisman J. (1976). A cell line (SCABER) derived from squamous cell carcinoma of the human urinary bladder.. Int J Cancer.

[OCR_00607] Pignatelli M., Smith M. E., Bodmer W. F. (1990). Low expression of collagen receptors in moderate and poorly differentiated colorectal adenocarcinomas.. Br J Cancer.

[OCR_00610] Plantefaber L. C., Hynes R. O. (1989). Changes in integrin receptors on oncogenically transformed cells.. Cell.

[OCR_00617] Rasheed S., Gardner M. B., Rongey R. W., Nelson-Rees W. A., Arnstein P. (1977). Human bladder carcinoma: characterization of two new tumor cell lines and search for tumor viruses.. J Natl Cancer Inst.

[OCR_00620] Rigby C. C., Franks L. M. (1970). A human tissue culture cell line from a transitional cell tumour of the urinary bladder: growth, chromosone pattern and ultrastructure.. Br J Cancer.

[OCR_00628] Schreiner C., Fisher M., Hussein S., Juliano R. L. (1991). Increased tumorigenicity of fibronectin receptor deficient Chinese hamster ovary cell variants.. Cancer Res.

[OCR_00633] Stamp G. W., Pignatelli M. (1991). Distribution of beta 1, alpha 1, alpha 2 and alpha 3 integrin chains in basal cell carcinomas.. J Pathol.

[OCR_00636] Terpe H. J., Tajrobehkar K., Günthert U., Altmannsberger M. (1993). Expression of cell adhesion molecules alpha-2, alpha-5 and alpha-6 integrin, E-cadherin, N-CAM and CD-44 in renal cell carcinomas. An immunohistochemical study.. Virchows Arch A Pathol Anat Histopathol.

[OCR_00643] Tomita Y., Nishiyama T., Fujiwara M., Sato S. (1990). Immunohistochemical detection of major histocompatibility complex antigens and quantitative analysis of tumour-infiltrating mononuclear cells in renal cell cancer.. Br J Cancer.

[OCR_00652] Tomita Y., Watanabe H., Kobayashi H., Nishiyama T., Tsuji S., Imai K., Abo T., Fujiwara M., Sato S. (1993). Expression of intercellular adhesion molecule-1 on transitional cell cancer. Possible significance in immunity against tumor cells.. Am J Pathol.

[OCR_00668] Witkowski C. M., Rabinovitz I., Nagle R. B., Affinito K. S., Cress A. E. (1993). Characterization of integrin subunits, cellular adhesion and tumorgenicity of four human prostate cell lines.. J Cancer Res Clin Oncol.

[OCR_00674] Zutter M. M., Mazoujian G., Santoro S. A. (1990). Decreased expression of integrin adhesive protein receptors in adenocarcinoma of the breast.. Am J Pathol.

